# Revisiting Premolars Using Cone-Beam Computed Tomography Analysis and Classifying Their Roots and Root Canal Morphology Using Newer Classification

**DOI:** 10.7759/cureus.38623

**Published:** 2023-05-06

**Authors:** Shreya Khanna, Leena Jobanputra, Jahnvi Mehta, Akshali Parmar, Aarti Panchal, Foram Mehta

**Affiliations:** 1 Conservative Dentistry and Endodontics, Government Dental College and Hospital, Jamnagar, Jamnagar, IND

**Keywords:** cone-beam computed tomography, variations, classification, canal configuration, premolars

## Abstract

Objective: The objective is to compare the internal morphology of premolars while applying the Vertucci and recent classification system for root canal variations in the Gujarat population using CBCT.

Materials and methodology: A sample size of 537 CBCT images collected from various diagnostic centers in Gujarat was analyzed. The root canal morphology was then classified by using two methods - Ahmed et al. and Vertucci classification system. Fisher’s exact test and Chi-square test were used for statistical analysis.

Results: All the premolars revealed a varied canal configuration. More than half of maxillary first and 42% of maxillary second premolars were double rooted. Vertucci type IV classification was the most common in first maxillary premolars and Type I and type IV were commonly seen in second premolars. According to the new system, the code ^2^N B^1^ P^1^ was commonly seen for first maxillary premolars. The majority of mandibular premolars were single rooted. Classification wise type I Vertucci and ^1 ^N^1 ^ were the most common types observed.

Conclusion: Premolars - both maxillary and mandibular - in this subpopulation had a wide range of root canal anatomical variations. Clinicians should be aware of this for a successful treatment outcome. The new system for classifying canal morphology describes the root and canal configurations in a more accurate and practical manner compared to the Vertucci classification and hence can be used routinely.

## Introduction

A successful root canal treatment necessitates precise knowledge and characterization of both usual and unusual canal anatomy [1]. Continued advancements related to magnification, micro-computed tomography (micro-CT), and cone-beam computed tomography (CBCT) have led to the recognition of the increasingly enormous range of anatomical variations in root canals with an attempt to receive the best possible clinical outcome [2].

The system proposed by Vertucci et al. has been the most frequently used classification and has been of use when categorizing many canal configurations. However, this classification fails to describe the number/configuration of the root(s) in maxillary premolars. In addition, and with an increasing range of imaging methods being used, many previously unreported anatomical complexities are being identified. As an attempt to provide a logical and simple solution, a new system for classifying root and canal morphology was proposed recently, which provides detailed information on tooth notation, number of roots, and root canal configuration. These anatomical variations have been precisely described by a new classification system introduced by Ahmed et al. in an exact, uncomplex, and reliable manner, allowing to classification of such root canals which were termed non-classifiable earlier that can be used clinically [3-5]. This new classification has codes for three separate components: the tooth number, the number of roots, and the root canal configuration [3-5]. The tooth number (N) can be written using any numbering system. The number of roots (R) is added as a superscript before the tooth number (RN). The type of root canal configuration in each root will be written as a superscript number(s) after the tooth number and will define the course of the root canal system beginning from the orifice(s) (O), through the canal (C) to the foramen (foramina) (F) as ^1^N^1-2^, ^2^ N^1^ B^1^ L^1^, and ^2^N^1^ M^1^ D^1^ [3]. Premolars - both maxillary and mandibular - have complexities in their anatomy, often being a cause of endodontic treatment failure [6-18]. CBCT is a non-invasive technology that allows a three-dimensional evaluation of root and canal morphology.

To date, there has been no detailed examination of the roots and canal systems of maxillary and mandibular first and second premolars while using the new classification. This study aims to investigate the number of roots, and root canal configurations using two systems for classifying root canal morphology in premolars in Gujarat population using CBCT.

## Materials and methods

A total of 1,200 CBCT images were collected from four diagnostic centers (Digident) in Gujarat (Jamnagar, Surat, Ahmedabad, and Patan) between October 2022 and March 2023. The images were collected from patients who had undergone CBCT scanning for various diagnostic reasons. Images were taken using a Care stream 8100 3DCBCT machine using parameters of voxel size 75microns, Fov 5×5 cm, 2 to 15mA, 60 to 90 KV for 15 sec at the exposure of 228 to 838 mGy.cm^2^. The identities of the patients were not revealed and only the patient’s age and tooth position in the arch were recorded. The study was approved by the MP Shah Government Medical College Ethics Committee. In all 537 CBCT images from a total of 1200 CBCT were assessed which met the following inclusion criteria: age 16-65 years; absence of periapical lesions, resorption, or canal calcification; no history of root canals; fully mature (closed) root apices; and good-quality CBCT images. Teeth with root caries, previous root canal treatment, immature apices, or root resorption were excluded.

Radiographic evaluation

The CBCT images were analyzed using CS 3D imaging software and were evaluated retrospectively by two experienced examiners. The CBCT images were numbered and evaluated separately to avoid any potential sources of bias. The data reliability was ensured. They were evaluated according to the following criteria: several roots and root canal configurations according to Vertucci and Ahmed et al classification system.

Statistical analysis

The statistical tests used were Fischer’s exact and Chi-square tests to compare the prevalence of a number of roots and canal configurations in maxillary and mandibular premolars. A difference was considered to be of statistical significance if the value of P ≤0.05 was seen.

## Results

Number of canals

In maxillary premolars, 24% of canals showed one canal at the apex whereas the majority as high as 75.1% showed two canals at the apex. Of the 125 studied maxillary second premolars, 58.4% had one root canal at the apex and 41.6% had two root canals at the apex. The majority of the mandibular premolars showed one canal in this study. 86.5% of mandibular first premolars and 97.7% of mandibular second premolars had a single canal at the apex (Table 1).

**Table 1:** Number of canals at apex

Arch	Tooth	Single canal at apex, n (%)	Two canals at apex, n (%)	Total, n (%)
Maxillary	First Premolar	34 (24.8)	103 (75.18)	137 (100)
	Second Premolar	73 (58.4)	52 (41.6)	125 (100)
Mandibular	First Premolar	116 (86.5)	18(13.43)	134 (100)
	Second Premolar	131 (97.7)	3(2.2)	134 (100)

Number of roots

More than half of maxillary first premolars had two roots whereas 42% of maxillary second premolars were double-rooted. Most of the mandibular premolars were single rooted. On statistical evaluation, a significant association was seen for maxillary premolars and their number of roots whereas for mandibular and number of roots (p<0.05); results came out to be highly significant (p<0.001) (Table 2).

**Table 2:** Number of roots Statistically significant: Fisher’s exact test *P≤0.05 (significant)

Arch	Tooth	Single rooted, n (%)	Double rooted, n (%)	Total, n (%)	P-value
Maxillary	First Premolar	45 (32.8)	92 (67.1)	137 (100)	0.049^*^
	Second Premolar	72 (57.6)	53 (42.4)	125 (100)	
	Total	117 (44.6)	145 (55.3)	262 (100)	
Mandibular	First Premolar	128 (95.5)	06 (4.47)	134 (100)	<0.001^*^
	Second Premolar	134 (100)	0	134 (100)	
	Total	262 (97.7)	6 (0.003)	268 (100)	

Root canal morphology 

*Maxillary First Premolars* 

The majority of maxillary first premolars had type IV Vertucci canal configuration (65.6%) followed by type 2, type 1 and type 3 (Table 3).

**Table 3:** Vertucci classification Statistically significant: Chi square test; *P≤0.05 (significant)

Arch	Tooth	Type I, n (%)	Type II, n (%)	Type III, n (%)	Type IV, n (%)	Type V, n (%)	Type VI, n (%)	Unique, n (%)	Total, n (%)	P-value
Maxillary	First Premolar	11 (8.02)	12 (8.7)	11 (8.02)	90 (65.6)	4 (2.9)	6 (4.3)	3 (2.1)	137 (100)	0.03^*^
	Second Premolar	44 (35.2)	14 (11.2)	15 (12)	40 (32)	5 (4)	7 (5.6)	0	125 (100)	
	Total	55 (20.9)	26 (9.9)	26 (9.9)	130 (49.6)	9 (3.4)	13 (4.9)	3 (1.1)	262 (100)	
Mandibular	First Premolar	102 (76.1)	4 (2.9)	10 (7.4)	4 (2.9)	4 (2.9)	10 (7.4)	0	134 (100)	<0.001^*^
	Second Premolar	124 (92.5)	4 (2.9)	3 (2.2)	0	0	3 (2.2)	0	134 (100)	
	Total	226 (84.3)	8 (2.9)	13 (4.8)	4 (1.4)	4 (1.4)	13 (4.8)	0	268 (100)	

Three out of 137 maxillary first premolars had shown unique variation in the form of 2-1-3 configuration and two of them had 2-1-2 configuration which could not be classified with Vertucci classification. In new classification system, ^2^N B^1^P^1^ was most prevalent (69.34%). All the teeth were easily classified in Table 4.

**Table 4:** Ahmed et al. classification for maxillary premolars

Tooth	^1^N^1^, n (%)	^1^N^1-2-1^, n (%)	^1^N^2-1^, n (%)	^1^N^1-2 ^, n (%)	^1^N^2 ^, n (%)	^2^NB^1^ P^1^, n (%)	^2^N^2-1^, n (%)	^2^N^2^, n (%)	Total, n (%)	P-value, n (%)
First Premolar	10 (7.2)	17 (1.2)	12 (8.7)	3 (2.1)	0	95 (69.3)	0	0	137 (100)	<0.001^*^
Second Premolar	43 (34.4)	15 (12)	0	12 (9.6)	2 (1.6)	37 (29.6)	14 (11.2)	2 (1.6)	125 (100)	
Total	53 (20.2)	72 (27.4)	12 (4.5)	15 (5.7)	2 (0.7)	132 (50.3)	14 (5.3)	2 (0.7)	262 (100)	

Maxillary Second Premolars

Type 1 Vertucci classification was most commonly observed (35.2%) followed by type 4 which was 32% (Table 3). Most of the second maxillary premolars were coded as ^1^N^1^ (34 %) followed by ^2^N B ^1^P^_1_^ (29.83%) and others (Table 3). Maxillary first premolars showed more variations in comparison to maxillary second premolar.

Mandibular First Premolars

Most prevalent canal variation was found to be type 1 according to Vertucci classification which was 76.11% followed by type 3 and type 6, both of which had prevalence of 7.46%. In accordance with Ahmed et al. classification ^2^N^1^ was most prevalent with 75.18% followed by ^1^N ^1-2 ^(9.77%) (Table 5).

**Table 5:** Ahmed et al. classification for mandibular premolars

Tooth	^1^N^1 ^, n (%)	^1^N^1-2-1^, n (%)	^1^N^2-1^, n(%)	^1^N^1-2^, n (%)	^1^N^2^, n (%)	^2^NB^1^L^1^, n (%)	^2^N^2-1^, n (%)	^2^N^2^, n (%)	Total, n (%)	P-value
First Premolar	100 (74.6)	10 (7.4)	4 (2.9)	14 (10.4)	0	6 (4.4)	0	0	134 (100)	<0.001^*^
Second Premolar	124 (92.5)	3 (2.2)	4 (2.9)	3 (2.2)	0	0	0	0	134 (100)	
Total	224 (83.5)	13 (4.8)	8 (2.9)	17 (6.3)	0	6 (2.2)	0	0	268 (100)	

Mandibular Second Premolar

Most prevalent canal variation was found to be type 2 (92.53%). ^1^N^1^ configuration was most prevalent here (92.53%) followed by others.

## Discussion

Knowledge pertaining to root canal morphology is one of the most valuable tools for the clinician in their arsenal when planning to treat an endodontic procedure. This, which can be provided precisely by CBCT along with a practical, reliable, and accurate classification system further aids the treatment [2]. A disproportionate number of records of subjects depict flare-ups and/or failures if the entire root canal system is not found and adequately managed. For the identification and successful treatment of teeth with various anatomical differences, an elaborate insight into root canal anatomy, careful radiographic analysis, and proper adjustment of the traditional access cavity preparation are pertinent. Before initiating root canal care, it is important to consider the possibility of root canal morphology variations. The effectiveness of root canal care is based on a meticulous understanding of both normal and abnormal root canal anatomy which can be effectively guided by CBCT. Clinical knowledge of such information is needed for proper shaping, cleaning, and obturation of the root canal system in three dimensions. The occurrence of missing roots or canals in teeth that required retreatment was 42%, according to Hoen and Pink. The current report has revealed enormous variations in the anatomy of the root and root canal of premolars among a Gujarati subpopulation. Since root canal configurations have a direct impact on the results of root canal treatment, dentists must be aware of the anatomical complications that exist. Missed canals, perforations, and canal transportations are all examples of iatrogenic operational errors caused by a lack of understanding of root canal morphology. The current study used CBCT to revisit premolars.

All the premolars, both maxillary and mandibular, first and second presented with varied anatomy, some in accordance with previously conducted studies and some showing different results. Single roots, two roots, and tree roots have been identified in maxillary first premolars, with the number of canals ranging from one to three per root [9,10]. In this study out of all the canals in the maxillary first premolar, a single canal at the apex was seen in 24%of the cases whereas, 75.1% exited in two separate foramen. Almost all canal variations were seen in maxillary first premolars with the highest of Vertucci type IV - 65.69%, type II - 8.75%, type III and type I at 8.02%, and others. This also showed the presence of unique canal variation in three teeth (2.18%), which were not classifiable using the Vertucci classification. This result was similar to the study done by Dinkar et al. where almost all Vertucci canal variations were seen except type VII and type IV being the predominant one [9,10]. Other studies also showed a lot of variations with respect to this tooth [6,7,11].

Of the 125 studied maxillary second premolars, 58.4% had one root canal at the apex and 41.6% had two root canals at the apex. They showed a higher probability of single root as compared to double as also reported by others [7,11]. Concerning the canal morphology, 35.2% of the teeth exhibited Vertucci type I configuration followed by type IV pattern (32%). This was different from the results reported by Raj et al. where type II configuration was predominant but similar to the one done by Mashyakhy et al. [11,14].

Mandibular first premolars were found to be single-root (94%) majorly and all mandibular second premolars were single rooted. Similar statistics showing a higher probability of single root but more percentage of double rooted as compared to this study have been reported by others [12,13,15]. On average, 91.0% of mandibular second premolars have a single canal and 9.0% have two or more canals [17]. Mandibular premolars with two or more canals were observed in 13.7% of American people and 46% of the Chinese population [18]. Multiple canals have been identified in mandibular first premolars from 0.2% to 39.5% [15-21]. This study found that 13% of mandibular first and 2% of mandibular second premolars had two canals, which is similar to the findings of Llena et al. [19] and Shetty et al. [20]. Conversely, for the present study, Sert and Bayirli [21] observed a greater incidence of two canals in mandibular second premolars, accounting for 29% of their research.

In the mandibular first premolar, all canal variations were present except type VII and VIII. The result can be presented as type 1 (76.11%), type III and type VI (7.46%), and type I, II and IV had 2.98% which coincides with a study done by Atul et al. who reported type I (70%), type II (7.97%), type III (3.62%), type IV (2.89%), type V (17.395), and type VI (0.72%). In a study done using CBCT and micro-CT imaging, type I variety (Vertucci’s classification) was most commonly encountered followed by type V and type III. CBCT investigation also leads to type I as the most numerous; however, the second most common was type III followed closely by others [13].

In the mandibular second premolar, maximum teeth had type 1 configuration (92.53%). Others were - type II (2.98%), type III (2.23%), and type VI (2.23%). In an investigation by Khademi et al. after type, I, the next most frequent morphologies in both first and second premolars were type V and type IV [12].

According to the recent system for classifying root and canal morphology (Ahmed et al.), ^2^N B^1^P^1^ was most prevalent (68.6%) in maxillary first premolars. This was followed by ^1^ N^1-2-1^ and ^1^ N ^2-1 ^configuration while most of the second maxillary premolars were coded as ^1^N^1^ followed by ^2^N B ^1^ P^1^,^1^ N1^-2-1 ^and ^2 ^N^2-1^ .^2 ^N^1 ^was most prevalent in mandibular premolars followed by ^1^N^1-2^ and second premolars predominantly showed ^1^ N^1^ code.

This is one of the first studies that make use of this new coding system to define root and canal configurations in all the premolars. This proved to have several advantages. It presented a more precise presentation of the tooth anatomy. As an example, it is confusing whether a Vertucci type IV canal is present in either a single- or a double-rooted tooth. Second, all three-canalled maxillary premolars are coded as Vertucci type VIII with no clarity as to the number of roots. Third, even when the number of roots was described in some studies together with the Vertucci classification, this was considered insufficient because three-rooted maxillary premolars can present in two forms (i.e., two buccal roots and one palatal root or one buccal root and two palatal roots). The use of the new system allowed root and canal morphology as well as anomalies (root fusion type) to be combined in a single code thus providing more detailed information. This kind of detailed, consistent documentation will be of benefit for clinical reports in which the reader can easily follow the anatomical landmarks of a given tooth in a systematic manner. Also, it is pertinent to have a knowledge of a number of roots as this has a direct implication on access opening, gauging the number and configuration of canals, further treatment, and reduce failures [22]. The use of this recent system allowed root and canal morphology to be amalgamated in a single code thus providing more elaborate information [4,5]. This study did not take into account any anomalies in the tooth and how it is to be classified which is the limitation of this study.

## Conclusions

Premolars have always proven to be a tooth with a wide range of root and canal anatomical variations which clinicians should be well acquainted with. They also need to know where canals merge or diverge to necessitate a favorable treatment outcome. More recently, development in digital dentistry, such as cone-beam and micro-computed tomography, and also magnification, have increased the number of reports on varied root canal anatomy. The recent system for classifying canal morphology describes the root and canal configurations in a more exact, reproducible, precise, and practical manner compared to the older ones. This system owning to its accuracy and practicality should be advocated to be used more in research, training, and clinical practice.
